# Pulmonary Edema Following Nifedipine Use in Pregnancy: A Case Report

**DOI:** 10.7759/cureus.68467

**Published:** 2024-09-02

**Authors:** Nour W Khattab, Ranim Razzouk, Bassem Tanios, Lara Nahouli, Pierre Bou Khalil, Nabil El Khoury

**Affiliations:** 1 Department of Internal Medicine, Division of Nephrology and Hypertension, American University of Beirut Medical Center, Beirut, LBN; 2 Department of Obstetrics and Gynecology, American University of Beirut Medical Center, Beirut, LBN; 3 Department of Internal Medicine, Division of Pulmonary and Critical Care Medicine, American University of Beirut Medical Center, Beirut, LBN

**Keywords:** tocolytic, pulmonary edema, pregnancy, nifedipine, antihypertensive

## Abstract

Calcium channel blockers are generally considered safe for use during pregnancy. They have several indications, including second-line therapy for lowering blood pressure and tocolytic therapy. We present the case of a 24-year-old woman, G1P0, with a twin gestation at 22 weeks, who presented with acute respiratory distress. Her symptoms occurred shortly after starting nifedipine as tocolytic therapy. Investigations revealed signs of volume overload and pulmonary edema. Extensive cardiac and infectious workups were normal. Obstetrical ultrasound did not show any signs of ovarian hyperstimulation syndrome. Based on these findings, she was diagnosed with acute pulmonary edema following tocolytic therapy with oral nifedipine. Nifedipine was stopped, and intravenous furosemide was started, resulting in rapid clinical improvement. We are reporting this case to raise awareness of this rare but life-threatening adverse event associated with nifedipine use in pregnant patients.

## Introduction

Calcium channel blockers (CCBs) can be safely used in pregnancy and in lactating women for several indications [1]. They are utilized for the treatment of hypertension and arrhythmias and even as tocolytic therapy to delay delivery and prevent preterm labor [1].

According to a systematic review and meta-analysis by Haas et al. in 2012, CCBs and prostaglandin inhibitors had the highest probability of improving neonatal and maternal outcomes and delaying delivery [2]. Particularly, nifedipine and nicardipine are commonly used as tocolytic agents [3]^.^ However, despite its efficacy and general safety profile, nifedipine is not without potential adverse effects [3]. 

In this article, we will discuss a serious side effect of nifedipine that may particularly affect pregnant women. Understanding such adverse reactions is crucial for clinicians to ensure the safety and well-being of both the mother and fetus during treatment.

## Case presentation

A 24-year-old woman, G1P0 with twin gestation by in vitro fertilization at 22 weeks and no past medical history, presented to our emergency department for acute dyspnea, respiratory distress, and desaturation with SpO_2_ at 86%. Non-invasive ventilation was attempted at first but failed, so she was intubated and placed on mechanical ventilation. The patient was normotensive on presentation with blood pressure (BP) of 110/60 mm Hg. However, after intubation and initiation of sedation with midazolam, she developed hypotension with BP reaching 85/50 mm/Hg. Therefore, a decision was made to start vasopressors with norepinephrine, which was successfully tapered and discontinued after 16 hours.

History was pertinent for starting nifedipine three days prior to presentation as a tocolytic agent for premature labor. She was prescribed 30 mg daily on the first two days, and on the third day, a total of 120 mg was taken.

On presentation, lung examination showed evidence of decreased breath sounds on bilateral bases and bilateral crackles reaching mid-lung fields. There was no lower limb edema. Initial laboratory workup showed increased inflammatory markers (white blood cell (WBC) 38,000 /cu.mm, procalcitonin = 3.39 ng/ml, C-reactive protein (CRP) = 91.3 mg/l). No history of recent steroid intake other than a prednisone course that was taken till the 14th week of gestation was recorded. Therefore, tazocin, azithromycin, and vancomycin were initiated empirically. She had a hematocrit of 31, and blood urea nitrogen (BUN), creatinine, and cardiac enzymes were normal. The 12-lead electrocardiogram showed sinus tachycardia with a heart rate of 120 beats/minute, with no other abnormality. The fetal heart rhythms were normal, and the obstetrical ultrasound did not document any ascites.

**Figure 1:**
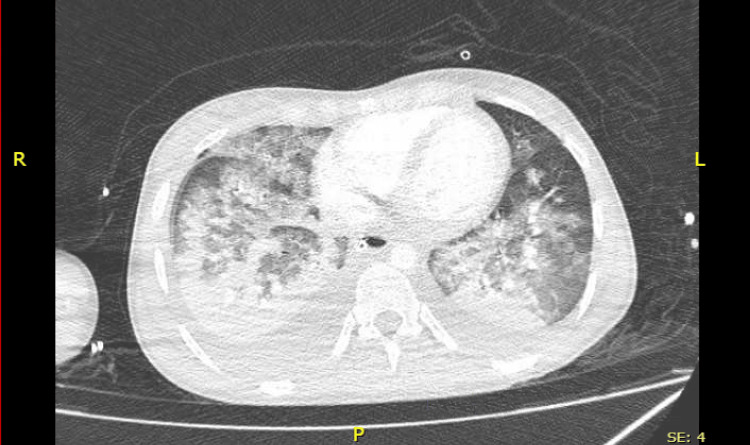
CT angiography showing evidence of pulmonary edema and bilateral moderate pleural effusions.

CT angiography was done and showed evidence of pulmonary edema and bilateral moderate pleural effusions. There was no evidence of pulmonary embolism and no ascites (Figure 1).

Transthoracic echocardiography showed a normal left ventricular size and geometry (left ventricle mass 109 g (77g/m^2^), normal left ventricular systolic function with an estimated left ventricular ejection fraction of 60-64%, and evidence of mild tricuspid regurgitation.

The initial tentative diagnosis was multilobar pneumonia/acute respiratory distress syndrome (ARDS) or diffuse alveolar hemorrhage (DAH). However, an extensive infectious workup, including broncho-alveolar lavage (BAL), was negative (Table 1). As for the hypotensive episode, it was most plausibly attributed to sedation and nifedipine; this subsequently resolved after the effect of these drugs had waned. DAH was excluded with BAL, in addition to negative anti-neutrophil cytoplasmic antibodies.

**Table 1:** Infectious workup

Bronchoalveolar lavage	Result
Bacterial culture	Negative
Fungal culture	Negative
Acid-fast bacilli smear and culture	Negative
Influenza virus A, influenza virus AHINI, rhinovirus, coronavirus OCA43, coronavirus 229E, coronavirus NL63, coronavirus HKU1, parainfluenza virus 1, parainfluenza virus 2, parainfluenza virus 3, parainfluenza virus 4, human metapneumovirus, bocavirus, Mycoplasma pneumonia, respiratory syncytial virus A/B, parechovirus, enterovirus, adenovirus, Staphylococcus aureus, Chlamydophila pneumoniae, Haemophilus influenza, Haemophilus influenza type B, Streptococcus pneumoniae, Pneumocystis jiroveci, Legionella lonbeachae/Legionella pneumophila, Klebsiella pneumoniae, Salmonella species, Moraxella catarrhalis, influenza C, Bordetella pertussis, COVID	Not detected

**Figure 2:**
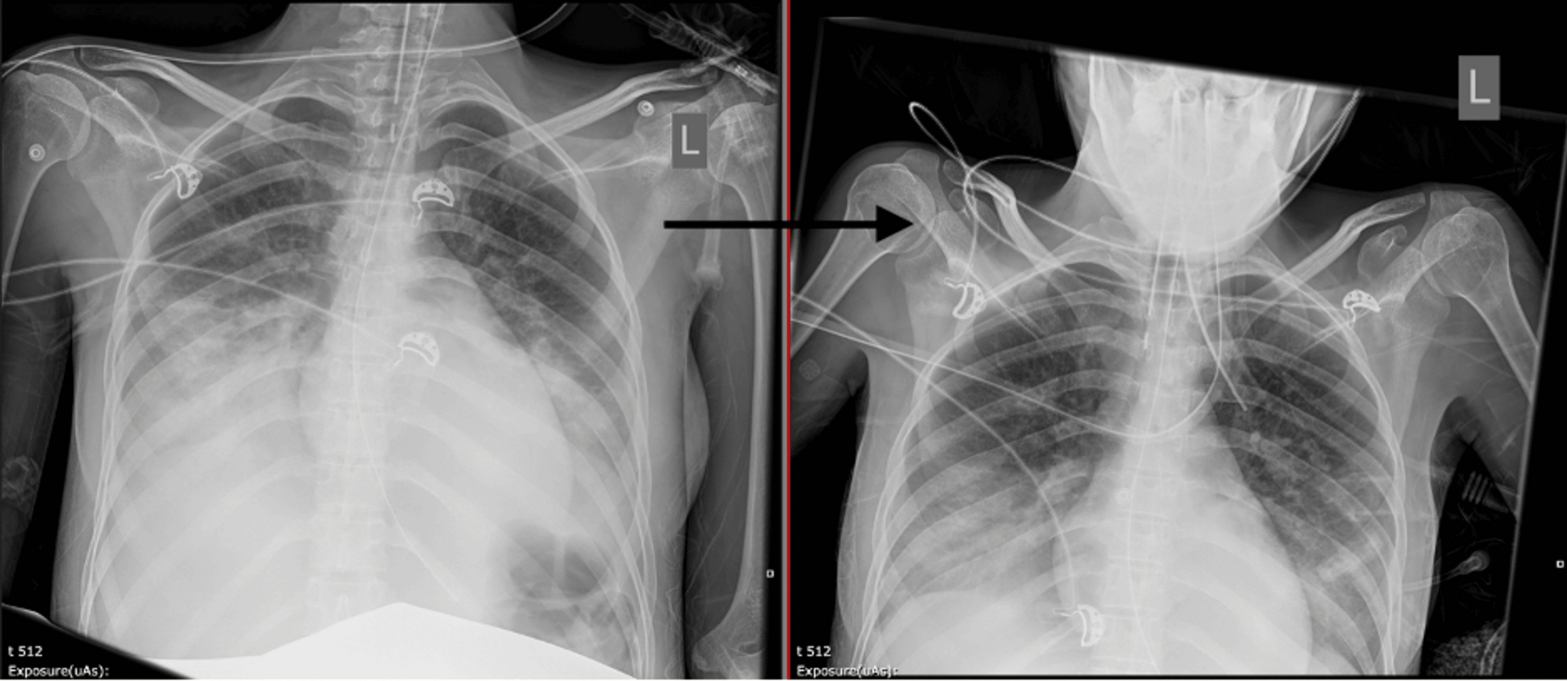
Chest X-ray on the right shows improvement in pulmonary edema and pleural effusion after diuresis.

The possibility of acute pulmonary edema secondary to CCB use was then entertained. Therefore, fluids were restricted, and the patient was initiated on intravenous furosemide, 40 mg daily for a total of four days, with improvement in her respiratory status and subsequent weaning off mechanical ventilation and extubation after 48 hours (Figure 2). The patient was discharged home with a viable gestation after eight days of admission. The patient was followed up as an outpatient, during which she experienced an uncomplicated pregnancy, delivered a healthy neonate at term, and had no long-term consequences or recurrences of pulmonary edema.

## Discussion

The use of dihydropyridine CCBs is widely accepted as a tocolytic therapy and has been used for this indication for more than 20 years [3,4]. This is due to the fact that CCBs have a significant impact on delaying labor by blocking voltage-gated L-type calcium channels (VGCCs) in the myometrium, which are crucial for uterine contractions [3]. Their appeal lies in their low cost, ease of administration, and oral preparation [4]. For the tocolytic use of nifedipine, it can be started with an initial dose of 20 to 30 mg orally, followed by 10 to 20 mg every three to eight hours, up to a maximum of 180 mg per day [3].

In addition to its use as tocolytic therapy, nifedipine is also considered a second-line therapy in the treatment of hypertension in pregnant women after labetalol and methyldopa [5]. The starting dose for the extended release is typically 30 or 60 mg once daily, which can be increased up to 90 mg daily, up to 120 mg per day [6]. A meta-analysis done by Ou et al. in 2023 showed that nifedipine may be the preferred option for treating acute severe hypertension during pregnancy compared to labetalol, as it exhibited similar therapeutic and safety effects while requiring less time to achieve target BP compared to labetalol [7].

The most frequent side effect observed with CCBs in pregnant women was headache [3]. Other side effects of CCBs are mainly due to excessive vasodilation and include hypotension, tachycardia dizziness, headache, flushing, myocardial infarction, hypoxia, elevated liver function tests, and lower extremity edema [4].

Acute pulmonary edema in pregnancy has an estimated rate of 0.08-1.5% [8]. Despite its rarity, it constitutes a life-threatening condition with high maternal and perinatal morbidity and mortality and therefore should not be ignored [8].

An increasing number of cases of acute pulmonary edema have been reported with the use of CCBs as tocolytic therapy in pregnant women [9,10,11]. This has also been observed with other tocolytic agents, especially beta-2 agonists. Obstetricians appear to be more familiar with this phenomenon than internists [12]. CCBs have also been reported to cause non-pregnancy-related pulmonary edema. This can occur in patients with severe primary pulmonary hypertension [13].

Several mechanisms for pulmonary edema secondary to nifedipine have been proposed. First, by exerting negative inotropic properties and causing vasodilation, nifedipine has the potential to trigger reflex tachycardia, ultimately resulting in diastolic dysfunction. This dysfunction increases left ventricular end-diastolic pressure, which in turn elevates left atrial and pulmonary venous pressures, ultimately leading to pulmonary edema. In pregnant patients, the already elevated circulatory volume can exacerbate this reduction in contractility, further increasing the risk of developing pulmonary edema [14].

Second, CCBs induce systemic vasodilation by inhibiting calcium influx into vascular smooth muscle cells, leading to a redistribution of fluid from the intravascular space to the interstitial space. In pregnancy, where plasma volume is already elevated, this fluid shift can overwhelm pulmonary capillaries, heightening the risk of pulmonary edema. In addition, CCBs may increase capillary permeability, further promoting fluid leakage into the pulmonary interstitium and alveoli. Moreover, the vasodilatory effect may be more pronounced in precapillary vessels than in postcapillary vessels, potentially elevating hydrostatic pressure and contributing to the development of pulmonary edema [7,9].

Furthermore, pregnant women are more susceptible to developing pulmonary edema due to physiological adaptations that occur during pregnancy [8]. These include a decrease in systemic and pulmonary vascular resistance, a reduction of colloid osmotic pressure by 30%, and activation of the renin-angiotensin-aldosterone system [8,15]. As a result, any increase in cardiac preload or permeability of the pulmonary capillaries will precipitate the risk of pulmonary edema in these patients [8].

Many factors may also contribute to the development of pulmonary edema in patients receiving a CCB. These include fluid retention from steroids, fluid infusion protocols, and the use of beta-agonists such as salbutamol [9]. Beta-adrenergic stimulation leads to an increase in cardiac output and activation of the renin-angiotensin-aldosterone system, vasodilation, and increased blood volume [15]. In addition, pre-existing cardiac conditions, such as hypertrophic cardiomyopathy, aortic stenosis, and pulmonary hypertension, have all been associated with an increased risk of acute pulmonary edema with the use of CCBs [16]. Pregnant patients with multiple gestations like our patient had a higher risk of acute pulmonary edema related to CCBs [17]. Some centers consider the use of CCBs to be relatively contraindicated in patients with multiple gestations [18]. In these cases, atosiban is considered a preferred tocolytic agent, because it has been shown to be effective with a low rate of side effects from 24 to 33 weeks of gestation [19].

In summary, the patient was diagnosed with pulmonary edema secondary to nifedipine. Cardiogenic origin was ruled out by normal echocardiography, EKG, and cardiac enzymes. Moreover, all infectious workups of possible ARDS induced by multilobar pneumonia were negative. Although the possibility of a concomitant lung infection is present, as evidenced by increased inflammatory markers on admission (CRP and procalcitonin), it would not explain the whole clinical picture of severe pulmonary edema. In addition, multilobar pneumonia and ARDS would not be expected to improve rapidly after initiating diuretics. 

Another possible diagnosis, ovarian hyperstimulation syndrome, was also considered. However, the lack of multisystem involvement with the sole involvement of the respiratory system, the absence of ascites, and the late onset of her presentation during pregnancy make this diagnosis less likely [20].

Furthermore, the rapid resolution of symptoms after stopping the offending medication and diuresis supports the diagnosis.

## Conclusions

This case highlights the potential for serious adverse effects associated with the use of nifedipine as a tocolytic agent in pregnant women, particularly the development of acute pulmonary edema. Despite its widespread use and general safety profile, nifedipine can induce pulmonary edema through mechanisms such as negative inotropic effects, systemic vasodilation, and increased capillary permeability, all of which are exacerbated by the physiological changes of pregnancy. This case underscores the importance of careful monitoring, especially in patients with multiple gestations or other risk factors for fluid overload. In addition, caution should be exercised when using nifedipine as an antihypertensive in pregnancy, as the same mechanisms may contribute to adverse outcomes. The rapid improvement in the patient's condition following discontinuation of nifedipine and the administration of diuretics further supports the diagnosis of nifedipine-induced pulmonary edema. Clinicians should remain vigilant for this potential complication and consider alternative tocolytic or antihypertensive agents in high-risk patients.
